# Evaluation of diffusion-weighted MRI and (18F) fluorothymidine-PET biomarkers for early response assessment in patients with operable non-small cell lung cancer treated with neoadjuvant chemotherapy

**DOI:** 10.1259/bjro.20190029

**Published:** 2019-07-20

**Authors:** Dominic Carlin, Alexander Weller, Gem Kramer, Yan Liu, John C Waterton, Arturo Chiti, Martina Sollini, A Joop de Langen, Mary E R O’Brien, Maria Urbanowicz, Bart KM Jacobs, Nandita deSouza

**Affiliations:** 1 CRUK Imaging Centre, The Institute of Cancer Research, Sutton, Surrey SM2 5NG, UK; 2 The Royal Marsden Hospital, Downs Road, Sutton, Surrey SM2 5PT, UK; 3 Department of Respiratory Diseases, VU University Medical Center, Amsterdam, The Netherlands,; 4 EORTC Headquarters, Brussels, Belgium; 5 Centre for Imaging Sciences, Division of Informatics Imaging & Data Sciences, School of Health Sciences, Faculty of Biology Medicine & Health, University of Manchester, Manchester Academic Health Sciences Centre, Oxford Road Manchester M13 9PL UK,; 6 Department of Biomedical Sciences, Humanitas University, Milan, Italy; 7 Nuclear Medicine, Humanitas Clinical and Research Centre, Milan, Italy; 8 Department of Thoracic Oncology, Netherlands Cancer Institute-Antoni van Leeuwenhoek Hospital, Amsterdam, The Netherlands,

## Abstract

**Objective:**

To correlate changes in the apparent diffusion coefficient (ADC) from diffusion-weighted (DW)-MRI and standardised uptake value (SUV) from fluorothymidine (^18^FLT)-PET/CT with histopathological estimates of response in patients with non-small cell lung cancer (NSCLC) treated with neoadjuvant chemotherapy and track longitudinal changes in these biomarkers in a multicentre, multivendor setting.

**Methods:**

14 patients with operable NSCLC recruited to a prospective, multicentre imaging trial (EORTC-1217) were treated with platinum-based neoadjuvant chemotherapy. 13 patients had DW-MRI and FLT-PET/CT at baseline (10 had both), 12 were re-imaged at Day 14 (eight dual-modality) and nine after completing chemotherapy, immediately before surgery (six dual-modality). Surgical specimens (haematoxylin-eosin and Ki67 stained) estimated the percentage of residual viable tumour/necrosis and proliferation index.

**Results:**

Despite the small numbers,significant findings were possible. ADC_median_ increased (*p* < 0.001) and SUV_mean_ decreased (*p* < 0.001) significantly between baseline and Day 14; changes between Day 14 and surgery were less marked. All responding tumours (>30% reduction in unidimensional measurement pre-surgery), showed an increase at Day 14 in ADC75^th^ centile and reduction in total lesion proliferation (SUV_mean_ x proliferative volume) greater than established measurement variability. Change in imaging biomarkers did not correlate with histological response (residual viable tumour, necrosis).

**Conclusion:**

Changes in ADC and FLT-SUV following neoadjuvant chemotherapy in NSCLC were measurable by Day 14 and preceded changes in unidimensional size but did not correlate with histopathological response. However, the magnitude of the changes and their utility in predicting (non-) response (tumour size/clinical outcome) remains to be established.

**Advances in knowledge:**

During treatment, ADC increase precedes size reductions, but does not reflect histopathological necrosis.

## Introduction

Non-small cell lung cancer (NSCLC) stage II and occasionally IIIA is routinely managed with surgery and adjuvant chemotherapy.^1^ Persistently poor survival rates^2^ have driven the use of induction (neoadjuvant) chemotherapy to reduce pre-surgical tumour burden^3–5^; results are favourable and equivalent to those where adjuvant chemotherapy is used.^6,7^ During neoadjuvant chemotherapy, response assessment relies on size-based (RECIST) measurements,^8^ which often only decrease 2–3 months after treatment initiation.^9^ Moreover, improved survival may be seen without a reduction in tumour size because of decreased metastatic propensity with cytostasis and post-treatment oedema.^9,10^ Therefore, early indicators of treatment response sensitive to biological changes in the tumour are desirable. Imaging techniques, such as diffusion-weighted MRI (DW-MRI) and PET, probe the tumour microenvironment and provide this potential.

DW-MRI provides an apparent diffusion coefficient (ADC), a biomarker of cellularity,^11^ which has been shown to increase in many cancers on treatment due to cell death.^12,13^ (18F)-fluorothymidine (FLT) uptake on PET, is also a promising biomarker for treatment response evaluation since its uptake is related to tumour cell proliferation.^14,15^ Some observational studies indicate that early reduction in FLT uptake correlates with later changes in tumour size^16–18^ others do not.^19^

This study was part of a larger programme validating imaging biomarkers for early response assessment in drug development.^20,21^ Neither ADC nor FLT uptake have been jointly evaluated with reference to histopathology in NSCLC: we hypothesised that they were related to pathological indices of response (ADC to necrosis and FLT Standardised Uptake Value (SUV) to proliferation) and correlated their changes in with histological measures of response (necrosis) and non-response (residual proliferative activity). We also studied longitudinal patterns of ADC and FLT-SUV change and linked them to RECIST-based size criteria for response and non-response. This international multicentre study was performed under quality-controlled conditions for multimodality, quantitative imaging^22,23^ to ensure comparable measurements on multiple scanners at multiple sites.

## Methods

### Patients

This prospective, multicentre, single-arm imaging trial (EORTC-1217) listed on clinical trials.gov (NCT02273271) recruited patients with confirmed clinical stage II-IIIA NSCLC eligible for curative intent surgery at three participating centres (Royal Marsden Hospital, UK; Humanitas, Milan; VUMC, Amsterdam, The Netherlands). 14 patients (nine male, five female) aged 53–78 years (mean 65.9 ± 7.1 years) gave written, informed consent (Table 1).

**Table 1.** Baseline characteristics of all non-small cell lung cancer (NSCLC) patients involved in the trial. Patient three withdrew consent prior to imaging and chemotherapy start

Patient	Gender	Age	Histopathology	Stage	Number of cycles	Platinum-based compound	Cytotoxic Compound	Reason for chemotherapy discontinuation
1	F	78	NSCLC	Stage IIIA	2	Carboplatin	Vinorelbine	Toxicity: lung infection and pulmonary embolism
2	M	71	NSCLC	Stage IIIA	3	Carboplatin	Gemcitabine	Toxicity: neutropenia
4	F	71	NSCLC	Stage IIIA	3	Carboplatin	Vinorelbine	Normal completion
5	M	57	NSCLC	Stage IIIA	3	Carboplatin	Vinorelbine	Normal completion
6	M	69	NSCLC	Stage IIA	3	Cis(1)/Carbo(2)	Vinorelbine	Normal completion
7	F	53	NSCLC	Stage IIIA	3	Cisplatin	Vinorelbine	Normal completion
8	M	68	NSCLC	Stage IIB	2	Cisplatin	Gemcitabine	Progressive disease
9	M	69	NSCLC	Stage IIIA	3	Carboplatin	Vinorelbine	Normal completion
10	M	74	NSCLC	Stage IIA	3	Carboplatin	Vinorelbine	Normal completion
11	F	60	NSCLC	Stage IIIA	3	Cis(2)/Carbo(1)	Gemcitabine	Normal completion
12	F	69	NSCLC	Stage IIIA	2	Cisplatin	Vinorelbine	Progressive disease
13	M	62	NSCLC	Stage IIIA	3	Cisplatin	Gemcitabine	Toxicity: thrombocytopenia
14	M	61	NSCLC	Stage IIIA	3	Cis(2)/Carbo(1)	Gemcitabine	Normal completion

**Figure 1.**
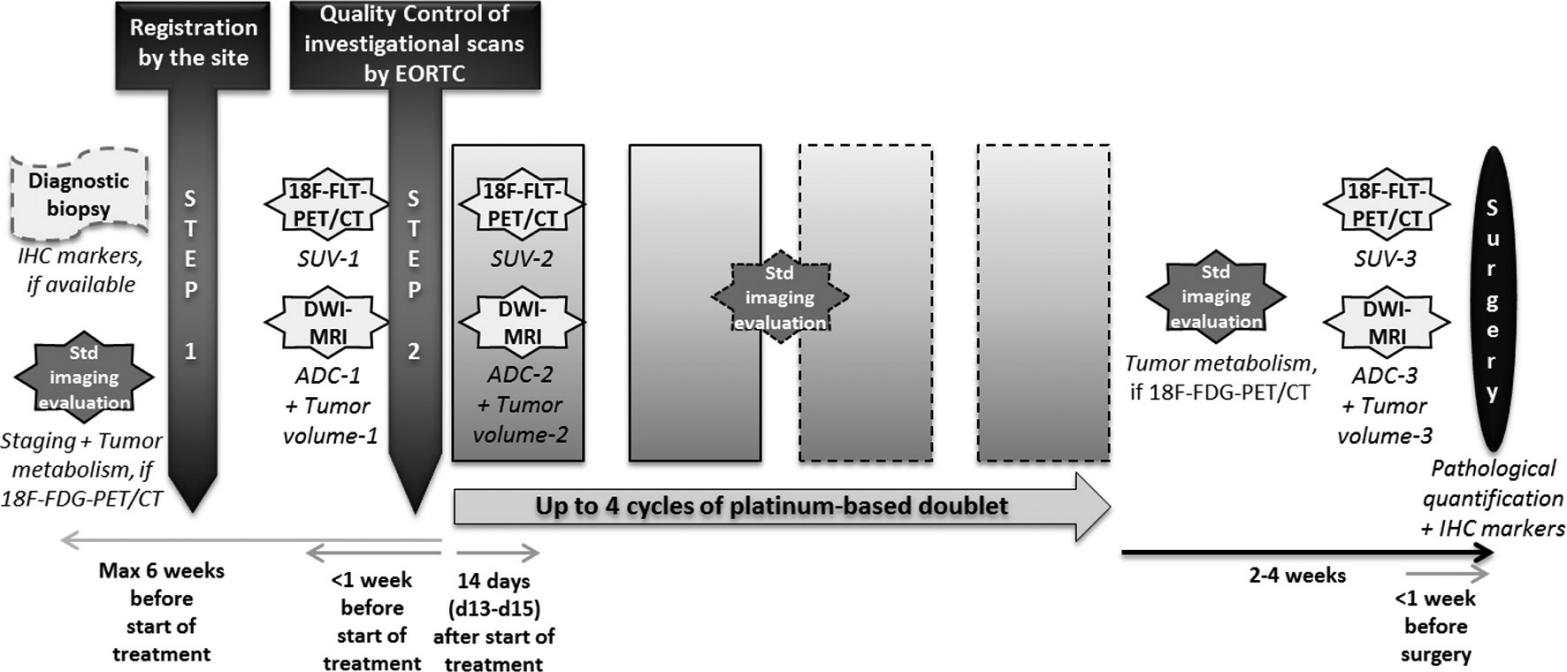
Design of this prospective, multicentre, single-arm imaging trial from registration to surgery.

Patients received platinum-based neoadjuvant chemotherapy without pemetrexed. They had DW-MRI and FLT-PET/CT between 1.10.2015 and 06.04.2017 at three time-points: i) baseline, before chemotherapy; ii) 14 days post-treatment and iii) within 1 week of surgery, 3–5 weeks after completing chemotherapy. Figure 1 summarises the study design.

### MRI

Patients were scanned on 4 clinical 1.5T MR platforms [Optima (GE Healthcare, Waukesha, WI); Avanto (Siemens AG, Erlangen, Germany); 2 Achieva (Philips Healthcare, Best, The Netherlands)] using phased-array body coils. All centres performed regular test-object scans for quality assurance (QA)/quality control (QC) and had previously participated in a technical validation phase to establish measurement reproducibility when performed in accordance with prescribed QA procedures.^24,25^ Axial *T*_1_ weighted turbo spin-echo in breath-hold and three-dimensional *T*_2_ weighted turbo spin-echo with variable flip-angle images were obtained as per local protocols.

DW-MRI was acquired during free-breathing using a single-shot echo-planar technique with short-tau inversion recovery fat suppression. Two blocks of 30 slices were acquired through the chest (thickness 5 mm, no gap), field-of-view 320 × 280 mm, four signal averages or four acquisitions, 3 b-values (100, 500, 800 s/mm^2^).

### Flt PET/CT

FLT was synthesised either in-house (Humanitas Clinical and Research Center, and VUMC) or by an external supplier (PETNET solutions Incorporated, Nottingham, UK). PET/CT was performed on a Discovery 690 (General Electric Healthcare, Waukesha, WI), Siemens Biograph 128m and Biograph 64 or Philips Gemini TF TOF 64 scanner in patients fasted for 4 h. Images were acquired without respiratory gating in a single bed position 60 min after injection of a 4MBq/kg FLT bolus. A non-contrast, non-breathhold CT scan was also obtained. PET/CT images were corrected for attenuation using low-dose CT scans according to EANM guidelines.^26^ Scanner accreditation through the EARL initiative (EANM Research Ltd; earl.eanm.org) guaranteed measurements’ reproducibility and comparability, across patient’s scans and among centers.

## Imaging biomarkers analysis

Each patient was imaged using the same scanner across all visits. Central review of images was performed. EARL approved reconstruction was used for PET/CT image analysis. ADC and SUV were calculated at baseline (ADC_1_, SUV_1_), Day 14 (ADC_2_, SUV_2_), and pre-surgery (ADC_3_, SUV_3_).

For DW-MRI, ROIs were manually segmented on computed *b* = 800 s/mm^2^ images with anatomical reference to T1-W, T2-W, ADC and *b* = 100 s/mm^2^ images on all slices with identifiable tumour. ROIs were copied onto corresponding ADC maps in Osirix. Whole tumour ADC histograms were generated. The median ADC (ADC_median_, primary endpoint), the 25^th^ and 75^th^ centiles and interquartile range (IQR) were recorded at each time-point. DWI-derived lesion volume at each time-point was estimated by multiplying total number of voxels by voxel dimensions.

All lesions were delineated using a 50% isocontour of the SUV_peak_ corrected for local background to derive tumour volumes of interest (VOI) designated as the proliferative volume. SUV was calculated within the VOI. SUV_mean_ (primary endpoint), SUV_max_, SUV_peak_ and total lesion proliferation (TLP, product of SUV_mean_ and proliferative volume) were recorded.

## Tumour response assessment

Percentage change of imaging biomarkers at Day 14 (∆ADC_early_, ∆FLT_early_) and pre-surgery (∆ADC_post_, ∆FLT_post_) in comparison to baseline values were calculated, where ∆ADC_early_ = 100%x(ADC_2_–ADC_1_)/ADC_1_, ∆SUV_early_ = 100%x(SUV_2_–SUV_1_)/SUV_1_, ∆ADC_post_ = 100%x(ADC_3_–ADC_1_)/ADC_1_ and ∆SUV_post_ = 100%x(SUV_3_–SUV_1_)/SUV_1_

Response between baseline and surgery was evaluated on CT images using longest uni-dimensional axis of the primary lesion defined by RECIST1.1 criteria (reduction in target lesion diameter >30%).^8^ Patients with appearance of new lesions who did not have further imaging were also classed as non-responders. The DWI and FLT images were not used to re-stage the patients, as this was outside the primary aim.

## Histopathology

Tumours were assessed on haematoxylin-eosin (HE) stained slides from surgical resection specimens by a central review pathologist (μ). Tumour surface area (mm^2^) was calculated on a maximum of 10 representative slides per lesion, along with areas of viable tumour, necrosis and fibrosis. Total tumour surface area was calculated by summing values from all slides; total viable tumour surface area and total necrotic tissue area were obtained similarly. The percentage of viable tumour or necrosis for each lesion was estimated from 100%x (total viable tumour surface area) or (total necrotic tissue surface area) respectively/(total tumour surface area).

Tumour cell proliferation was assessed by immunohistochemistry (Ki67 staining).^27^ Positive nuclei were calculated on each slide in at least 100 cells in the representative areas of the tumour and expressed as a percentage. The mean Ki67 index from all reviewed slides was the final sample score.

## Statistical considerations

Co-primary imaging endpoints were ΔADC_early_ defined on ADC_median_ and ΔSUV_early_ defined on SUV_mean_; alternative ADC and SUV measures were secondary endpoints. The primary pathology endpoint measures were percentage of viable tumour cells; percentage of necrosis and proliferative activity (Ki67 index) were secondary endpoints. Responder status was based on unidimensional size and included patients who did not undergo surgery. 31 lesions were needed to demonstrate with 95% confidence (one-sided) with 90% power that the absolute correlation between the imaging biomarker change and the pathological response was >0.5 (H0: rho ≤0.5) if the true correlation is 0.8 (H1: rho >0.5).

Statistical analysis used SAS9.4 (STAT14.3). Graphs were generated using Excel (version14.0.7212.5000) and validated in SAS. Correlations between continuous endpoints were assessed using the Spearman rank correlation test with Fisher transformation and assumed to be positive between ΔSUV_early_ and %viable tumour (ρ_0_ = 0.5), and negative between ΔADC_early_ and %viable tumour (ρ_0_ = −0.5). Equivalent two-sided 90% confidence intervals are reported to check futility.

The one-sided type I error was fixed at 1% for all secondary and exploratory analyses. When relevant, equivalent two-sided 98% confidence intervals are reported.

Longitudinal comparisons of ADC, SUV and volumes were analysed with a two-sided Wilcoxon signed-rank test.

## Results

14 patients were successfully screened and 13 finally participated (one withdrew). 13 patients had DW-MRI and^16^FLT-PET/CT at baseline (10 had both), 12 were re-imaged at Day 14 (eight dual-modality) and nine after completing chemotherapy, immediately before surgery (six dual-modality). four patients did not have surgery, three due to progressive disease and one due to toxicity. An overview of imaging scans and surgery status is presented in Table 2.

**Table 2.** Imaging and surgical status for individual participants

**Patient ID**	**Baseline DWI**	**Baseline FLT**	**Day 14 DWI**	**Day 14 FLT**	**Pre-surgical DWI**	**Presurgical FLT**	**Surgical sample**
1	√		√				
2	√	√	√		√	√	√
3	WITHDREW						
4	√	√		√		√	√
5	√		√		√		
6		√					
7	√	√	√	√	√	√	√
8	√	√	√	√			
9	√	√	√	√	√	√	√
10	√	√	√	√	√	√	√
11	√	√	√	√	√	√	√
12	√	√	√	√			
13	√	√	√	√		√	√
14	√	√	√	√	√	√	√

Of the 12 patients imaged at Day 14, three met the criteria for response of the primary lesion (30% reduction in unidimensional measurement) on CT performed prior to surgery (Table 3). Eight patients were classed as non-responders; the numbers of patients imaged at each time-point with each imaging modality are given in Table 4. The remaining patient was not included in the response assessment because toxicity to Carboplatin plus Vinorelbine prompted a change to the targeted agent Erlotinib.

**Table 3.** Change in long axis with treatment indicating three responders by RECIST criteria at the pre-surgical time-point

**Patient ID**	**Long axis baseline (mm**)	**Long axis Day 14** (**mm**)	**Change Day 14** (**%**)	**Long axis pre-surgery** (**mm**)	**Change pre-surgery** (**%**)
1	59	53	−10.1		
2	60	49	−18.3	50	−16.7
3					
4	33	30	−9.1	25	−24.2
5	41	40	−2.4	44	7.3
6					
7	36	43	19.4	32	−11.1
8	68	68	0		
9	43	42	−2.3	35	−18.6
10	56	55	−1.8	52	−7.1
11	63	40	−36.5	16	−74.6
12	71	69	−2.8	70	−1.4
13	70	58	−17.1	48	−31.4
14	73	54	−26.0	48	−34.2

**Table 4.** Median and (range) of primary lesion values of DW-MRI and FLT-PET metrics at baseline for all patients, responders and non-responders. Note: all patients for DW-MRI and FLT-PET each include a patient who did not have response assessed

**DW-MRI**	ADC_median_	ADC_25th_	ADC_75th_	ADC_IQR_
	(x10^−3^ mm^2^/s)	(x10^−3^ mm^2^/s)	(x10^−3^ mm^2^/s)	(x10^−3^ mm^2^/s)
**Baseline (*n* = 12**)	1.14	0.91	1.33	0.42
	(0.80–1.49)	(0.56–1.26)	(0.93–1.42)	(0.20–0.56)
*Responders (n = 3)*	0.89	0.74	1.07	0.33
	(0.82–1.29)	(0.72–1.09)	(0.93–1.59)	(0.20–0.50)
*Non-Responders (n = 8)*	1.16	0.91	1.36	0.46
	(0.80–1.49)	(0.56–1.26)	(1.05–1.73)	(0.22–0.56)
**FLT-PET**	SUV_max_	SUV_peak_	SUV_mean_	TLP
**Baseline (*n* = 11)**	6.21	4.78	3.54	141
	(4.9–14.0)	(3.1–12.0)	(2.4–8.2)	(38–2870)
*Responders (n = 3)*	11.2	9.23	6.74	379
	(9.9–14.0)	(8.4–12.0)	(6.7–8.2)	(141 – 522)
*Non-Responders (n = 7)*	5.62	4.72	3.11	95.3
	(4.9–11.7)	(3.1–9.9)	(2.4–7.6)	(38–2870)

Re-staging the patients with either DWI or FLT was outside the remit of the study. Also repeat mediastinoscopy was not routinely performed. Repeat CT scan pre-surgery detected a new lesion in one patient of our cohort, who was therefore classified as a non-responder.

## Correlation of imaging parameters and pathological measures

**Figure 2.**
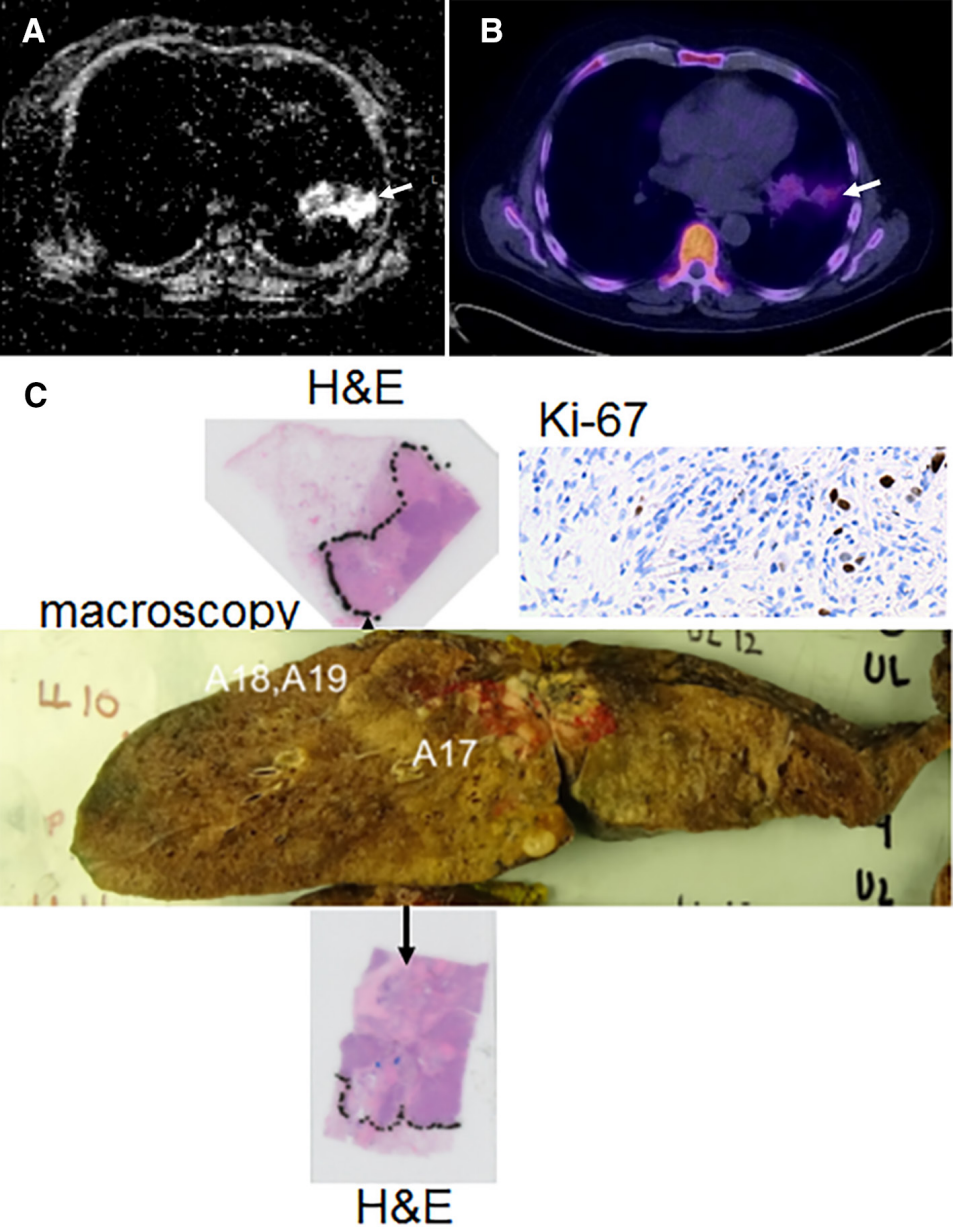
A 70-year-old patient with lung cancer treated with platinum based neoadjuvant chemotherapy: ADC map derived from DW-MRI prior to surgery (a), FLT-PET at the same time-point (b), show residual tumour with lateral consolidation (solid white arrows). Following resection, this was corroborated on the corresponding slice of the macroscopic surgical specimen (c, centre). Residual tumour was confirmed on H&E stained sections. Ki-67 stain shows the low level of proliferative activity (brown stain) within the centre of the tumour.

Early changes in imaging parameters were compared with pathological measures (Figure 2). In patients who underwent surgery and had imaging at Day 14 (*n* = 8), there was no meaningful correlation at the 1% significance level between ∆ADC_early_ and the %viable cells (*r* = 0.50, *p* = 0.99, 90% CI = [−0.30; 0.87]) or ∆SUV_early_ and the %viable cells (*r* = 0.07, *p* = 0.85, 90% CI = [−0.64; 0.71]). The two-sided confidence intervals did not reach the ρ = −0.8 and ρ = 0.8 respectively correlation as originally hypothesised. Similarly, there were no meaningful correlations between presurgical ADC_median_ and %necrosis (*r* = 0.26, *p* = 0.56, 98% CI = [−0.80; 0.92]). The observed correlation between Ki-67 and presurgical SUV_mean_ was higher than ρ = 0.8 as originally hypothesised (*r* = 0.86, *p* = 0.08, 98% CI = [0.05; 0.98]).

## Longitudinal patterns of response to treatment

**Figure 3.**
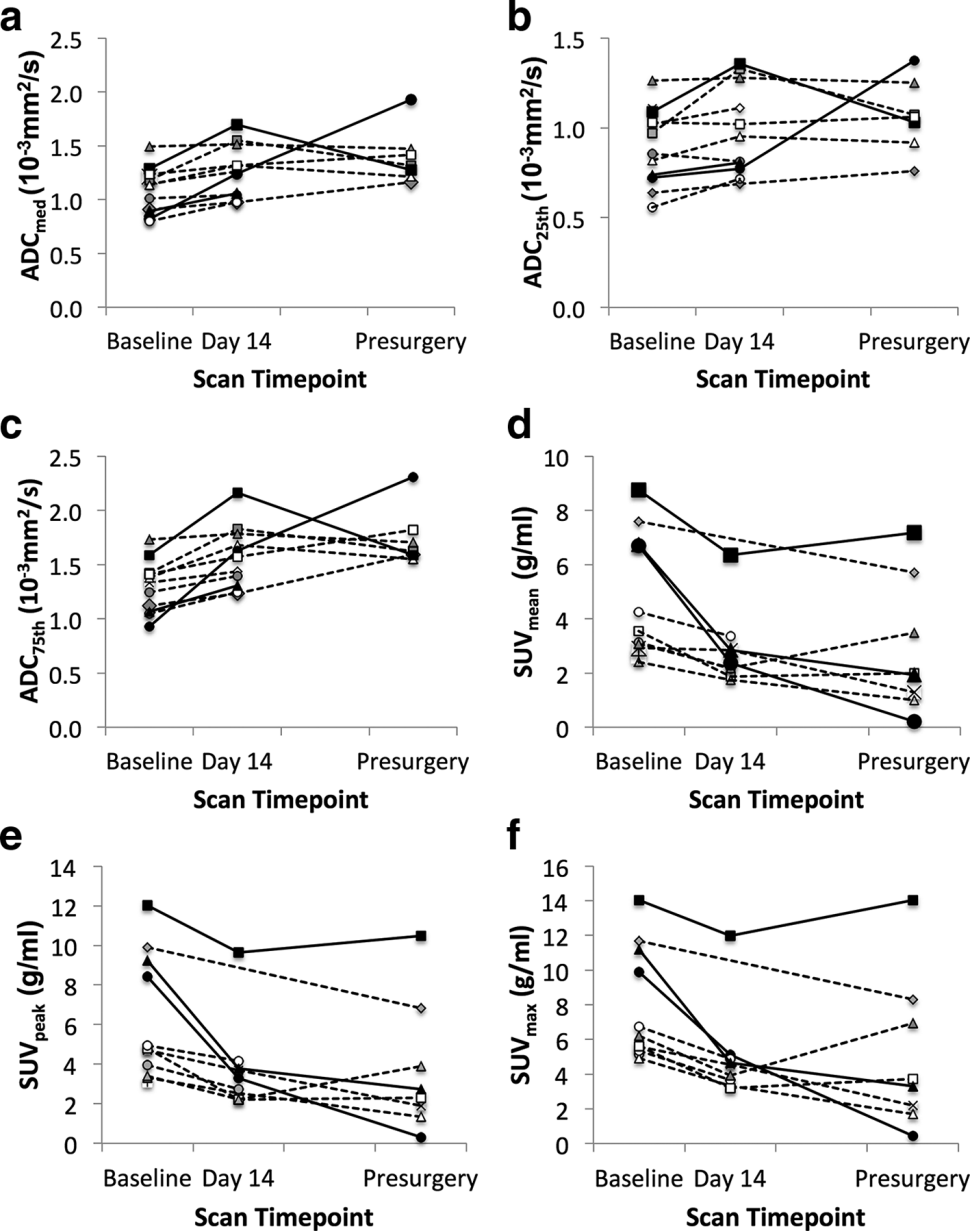
Absolute values of (a) ADC_median_, (b) ADC_25th_ and c) ADC_75th_ , (d) SUV_mean_, (e) SUV_peak_, and (f) SUV_max_ at each time-point (solid lines =responders, dashed lines=non-responders) show the increase in ADC values and decrease in FLT-SUV parameters with time on treatment.

Baseline ADC and SUV values from DWI and FLT imaging respectively at each time-point are presented in Table 4 **.** The relative changes in imaging metrics are presented in Table 5 for DWI and Table 6 for FLT. The ADC_median_ increased (*p* < 0.001) and SUV_mean_ decreased (*p* < 0.001) significantly between baseline and Day 14, however the change between Day 14 and surgery was less marked (Table 5), indicating that this parameter changes early. The changes in ADC_median_, ADC_75th_ and ADC_IQR_ at Day 14 relative to baseline were generally larger in responders than non-responders (Figure 3a–c) although small numbers precluded statistical evaluation. Early changes in ADC_25th_, however, were comparable between responders and non-responders.

**Table 5.** Mean ± standard deviation and range of percentage change of DWI metrics relative to baseline at Day 14 and within 1 week of surgery

	Mean ± SD ∆DWI %				
	(Range)				
	Volume	ADC_median_	ADC_25th_	ADC_75th_	ADC_IQR_
**Day 14**	−18.4 ± 30.8	18.2 ± 15.1	12.2 ± 13.1	22.6 ± 20.1	54.7 ± 90.3
All patients (*n* = 11)	(-86.6–29.2)	(1.4–51.1)	(-5.0–36.7)	(3.0–75.6)	(3.0–319.6)
**Pre-surgery**	−30.1 ± 41.7	27.7 ± 48.5	18.3 ± 32.7	35.0 ± 52.5	81.8 ± 126.7
All patients (*n* = 7)	(-99.8–39.6)	(-1.4–135.3)	(-5.4–90.0)	(-1.4–148.7)	(-2.4–356.9)

**Table 6.** Mean ± standard deviation and range of percentage change of FLT metrics relative to baseline at Day 14 and within 1 of surgery

	Mean ± SD ∆FLT				
	(Range)				
	Volume	SUV_max_	SUV_peak_	SUV_mean_	Total lesion proliferation
**Day 14**	−21.0 ± 38.6	−35.0 ± 13.9	−28.4 ± 33.1	−35.1 ± 18.9	−49.5 ± 23.1
All patients (*n* = 9)	(-68.0–55.2)	(-58.3–−14.7)	(-61.1–47.9)	(-65.7–−3.0)	(-89.7–−18.4)
**Pre-surgery**	−19.6 ± 41.0	−43.2 ± 37.0	−45.4 ± 34.7	−44.3 ± 34.9	−52.8 ± 32.9
All patients (*n* = 8)	(-91.7–69.2)	(-95.6–11.9)	(-96.3–14.4)	(-97.0–13.6)	(-98.9–−4.7)

The decreases in SUV_mean,_ SUV_max_, SUV_peak_ and TLP at Day 14 relative to baseline were generally larger in responders than non-responders (Figure 3d–f); again small numbers precluded statistical evaluation.

## Changes with respect to measurement repeatability

**Figure 4.**
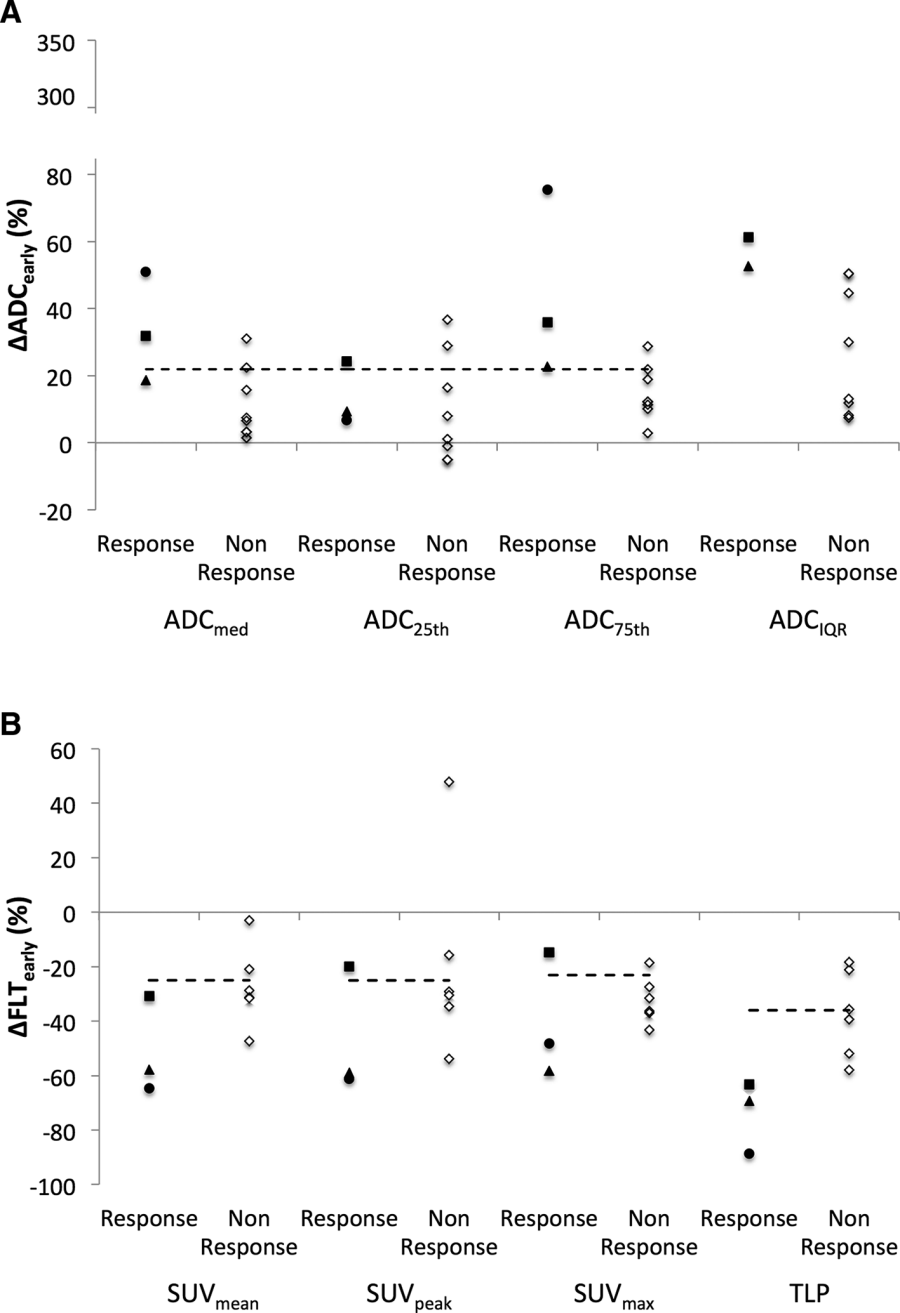
Comparison of change at Day 14 of treatment relative to baseline of a) DWI and b) FLT metrics for responders and non-responders with respect to measures of repeatability (dashed lines). Responders are shown with black filled markers and the y-axis for ∆ADC_early_ has been broken to include a patient with high ∆ADC_IQR_. The increase in ADC_75_ and ADC_IQR_ and decrease in FLT-TLP was outside the limits of measurement variability in responders.

Changes in imaging biomarkers at Day 14 were also assessed with reference to previously established test-retest estimates of measurement repeatability in order to exclude changes that represent measurement variability.^24,25^ (Figure 4). Increases in ADC_median_ greater than the limits of measurement repeatability were seen in four patients (2/3 responders, 2/7 non-responders). The measurable increases in ADC_75th_ were seen on all three responders but only in 1/7 non-responders, while ADC_25th_ only increased in 1 responder and two non-responders. Measurable decreases at Day 14 were observed for SUV_mean_ in seven patients (all 3 responders and 4/6 non-responders) SUV_max_ (2/3 responders, 5/6 non-responders) and in six patients for SUV_peak_ (2/3 responders, 4/6 non-responders), and TLP (all 3 responders and 3/6 non-responders).

## Comparison of imaging volumes

**Figure 5.**
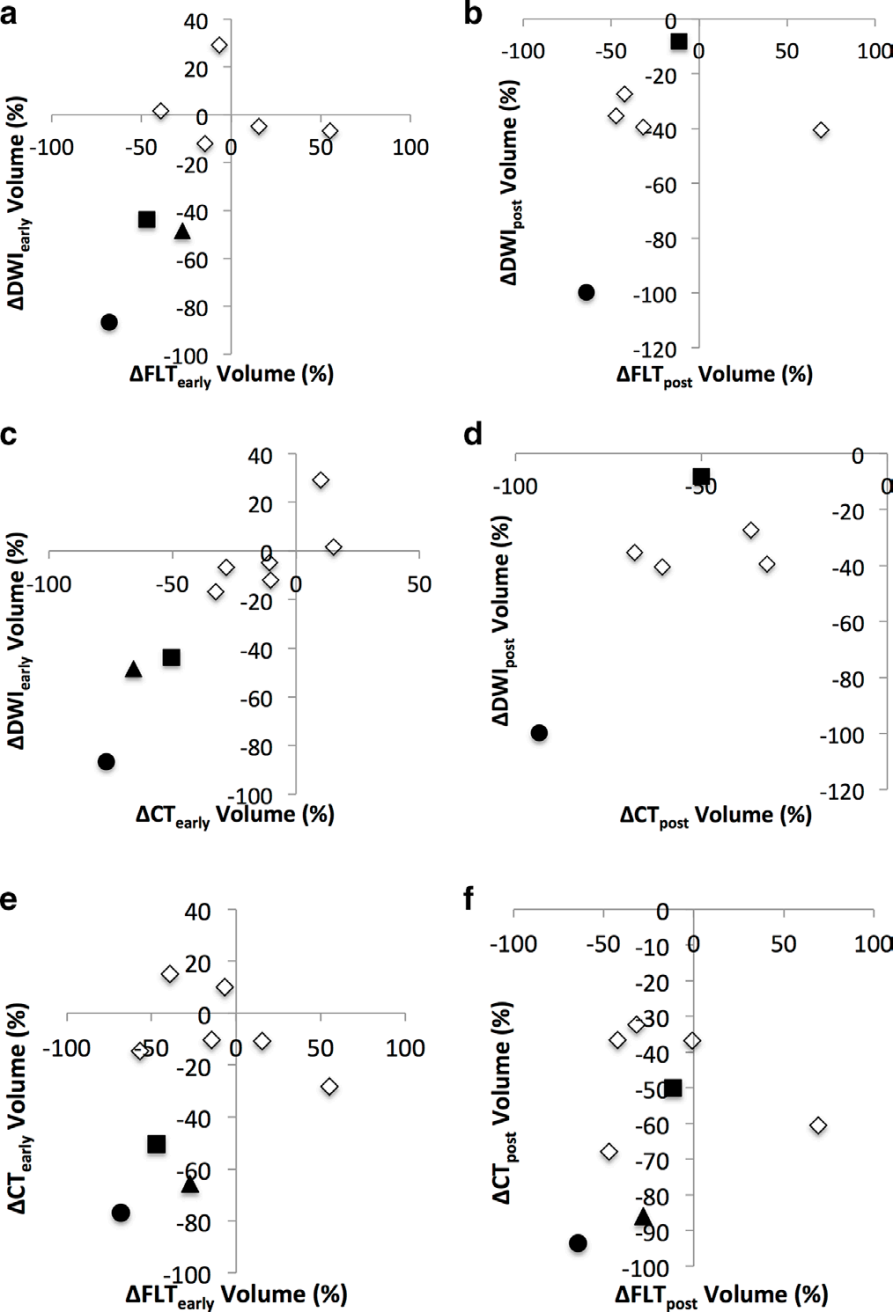
A comparison of changes at Day 14 (top row) and pre-surgery (bottom row) from baseline between tumour volumes derived from DWI and FLT (a, b), DWI and CT (c, d) and FLT and CT (e, f). Responders are represented by black filled markers (*symbols: square=patient 11, triangle=patient 13, circle = patient 14*).

Tumour volumes generally decreased between baseline and Day 14 for all imaging modalities, with mean ± standard deviation volume reductions of 20.9±33.8% for DWI (*n* = 9, *p* = 0.03), 21.0±38.6% for FLT (*n* = 9, *p* = 0.16) and 26.4±30.5% for CT (*n* = 10, *p* = 0.03); volume reductions between techniques were not significantly different at the 1% significance level. For patients in whom the pre-surgery scan was available, DWI (*n* = 7) and CT-derived (*n* = 8) tumour volumes showed average reductions of 41.7±30.8% for DWI and 58.0±23.3% for CT from baseline. The mean pre-surgical FLT volume reduction (*n* = 8) was 19.6±41.0% compared to baseline (*p* = 0.1). Comparative volume changes are illustrated in Figure 5.

There was no significant correlation between the ∆ADC_median_ at Day 14 and the DWI-assessed tumour volume change measured at Day 14 (*r* = −0.39, one-sided *p* = 0.68) or prior to surgery (*r* = −0.14, one-sided *p* = 0.93). There was also no significant correlation between the ∆SUV_mean_ at Day 14 and the FLT tumour volume change measured at Day 14 (*r* = −0.08, one-sided *p* = 0.95) or prior to surgery (*r* = 0.25, one-sided *p* = 0.75).

## Correlation of imaging techniques

There was no meaningful correlation between ADC_median_ and SUV_mean_ at baseline (*r* = −0.27, one-sided *p* = 0.78), Day 14 (*r* = −0.06, one-sided *p* = 0.88) or pre-surgery (*r* = −0.49, one-sided *p* = 0.55). There was also no significant correlation between ∆ADC_median_ and ∆SUV_mean_ (*r* = −0.24, one-sided *p* = 0.44).

## Discussion

This pilot study showed no significant association between changes in imaging biomarkers (ADC and FLT-SUV) and pathological measures of response.. Despite the small numbers, this is a robust finding because even the confidence intervals did not reach the correlations originally hypothesised. Therefore, it is unlikely that these imaging biomarkers relate to the pathological features as quantified here. It is also unlikely that we would have seen an association between the imaging parameters and histopathology had the planned sample size been achieved. The original study design did not include a futility analysis, but had a futility analysis been included, we could not have ethically justified continued recruitment in light of the poor response to neoadjuvant chemotherapy in these patients. Our primary pathological measure was the percentage of viable residual tumour, which may not have been the best histopathological endpoint, as pathological evaluation was semi-quantitative. Tumour cellularity derived from digital pathology analyses is a more robust measure that could be considered for future validation of imaging biomarkers on histopathology. Secondary analysis found a positive association between pre-surgical FLT-SUV and Ki-67 in our data, however the literature on the relationship between these two parameters is ambiguous. Positive correlations have been found between the two in gliomas,^28^ breast cancer^29^ and a mixture of lung nodules,^15^ but a number of studies in a variety of tumour types have found no relationship.^30–32^

Our exploratory analysis suggests that changes in imaging biomarkers (measured in a multicentre, multivendor setting against established reproducibility criteria) occur before unidimensional tumour size changes of >30%. ADC generally increased over time, which is linked to an increase in necrotic and apoptotic cell death induced by treatment.^33^ Consistent with previous studies, patients who responded to treatment in our sample generally had greater ADC_median_ increases at Day 14 relative to baseline, but statistical analysis was not relevant given the low sample size and small numbers of responders.^34,35^ In other NSCLC data, early (after 1 cycle of chemotherapy) increases in ADC have been associated with increased progression-free and overall survival^34^ and increased tumour volume reduction.^35^ In our data, responders also had larger early increases in ADC_75th_ than non-responders, which could reflect an increase in necrotic domains following treatment. Although relatively unexplored in NSCLC, changes in ADC histogram parameters have been associated with improved response in other tumour types.^36–38^

FLT-SUV generally decreased by Day 14 but changed inconsistently thereafter. Responding patients had amongst the highest FLT-SUV values at baseline, which is expected as chemotherapy targets proliferative cells. They also had greater early decreases in SUV_mean_ and TLP; again the small numbers precluded statistical analysis. A previous study (*n* = 9) assessing NSCLC treatment response using FLT-PET^19^ found that FLT parameters did not distinguish between responders and non-responders after 1 cycle of chemotherapy, so that the use of FLT-PET to indicate response remains debatable.

The use of test-retest metrics to establish measurement variability is critical, particularly in a multicentre setting where multiple scanner platforms add variability. Repeatability, (“closeness of the agreement between the results of successive measurements of the same measurand carried out under the same conditions of measurement”), can be represented by limits-of-agreement interval.^39^ Our previous test-re-test data from multiple centres including those in this study in larger series for ADC^24^ and FLT,^25^ gave repeatabilities of 22 and 25% respectively. Therefore, we are confident that the changes measured here exceed measurement variability. For some outcomes (such as ADC_75th_), changes were typically observed more often for patients classified as responders. Importantly, changes in imaging biomarkers preceded volumetric changes. If future research confirmed a strong association between (absence of) early change in the biomarker that remained within measurement variability, then these biomarkers could be used for early detection of (in)effective treatment.

NSCLC responded poorly to platinum-based neoadjuvant chemotherapy (four patients became inoperable). Unfortunately, response assessment based on size is confounded by peritumoral atelectasis and inflammation as these regions are often indistinguishable from residual tumour on CT. This is more problematic when segmenting volumes: CT volumes increased with treatment in two of the three patients classified as responders, likely caused by the inclusion of inflammatory tissue.

The main limitations of this study were firstly, the stringent inclusion criteria that required patients to be operable at the outset. The use of neoadjuvant chemotherapy in these cases represented a departure from standard-of-care and severely limited recruitment. Recruitment also was severely affected by 3 cases of disease progression on chemotherapy. Secondly, although the preclinical literature indicates that ADC is a biomarker of necrosis/apoptosis, in a clinical setting histopathological analysis is influenced by technical aspects and observer interpretation, so that the data is less reliable.^40^ Correlation of imaging biomarkers with histology is difficult at a whole lesion level because in large tumour specimens as here, selected sections may not be representative of the entire lesion. Image analysis of whole digitised pathology specimens would address these issues in future. Thirdly, progressive disease due to new metastases not increase in primary tumour size, confounded response classification in two cases. Finally, two patients withdrew consent for MRI either during or following MRI examination. The tolerability of multiple imaging examinations in patient groups with poor performance status and compromised respiratory function is an important consideration when planning future studies.

In conclusion, this study adds to the body of evidence documenting longitudinal changes in imaging biomarkers and indicates their lack of correlation with traditional histological markers of response and non-response. Changes in ADC and FLT following neoadjuvant chemotherapy in NSCLC occur as early as 14 days after initiating treatment and exceed measurement variability in responders. However, the utility of early changes in imaging biomarkers as well as the baseline biomarker levels in predicting (non-) response as defined by clinical outcome or tumour size remains to be established.
